# Controlled Cardiac Computed Tomography

**DOI:** 10.1155/IJBI/2006/12819

**Published:** 2006-04-11

**Authors:** Erwei Bai, Chenglin Wang, Ying Liu, Ge Wang

**Affiliations:** ^1^ Department of Electrical and Computer Engineering and Department of Radiology, University of Iowa, Iowa City, IA 52242, USA; ^2^ Controlled Cardiac CT, LLC, Iowa City, IA 52246, USA

## Abstract

Cardiac computed tomography (CT) has been a hot topic for years because of the clinical importance of cardiac diseases and the rapid evolution of CT systems. In this paper, we propose a novel strategy for controlled cardiac CT that may effectively reduce image artifacts due to cardiac and respiratory motions. Our approach is radically different from existing ones and is based on controlling the X-ray source rotation velocity and powering status in reference to the cardiac motion. We theoretically show that by such a control-based intervention the data acquisition process can be optimized for cardiac CT in the cases of periodic and quasiperiodic cardiac motions. Specifically, we formulate the corresponding coordination/control schemes for either exact or approximate matches between the ideal and actual source positions, and report representative simulation results that support our analytic findings.


Hindawi Publishing Corporation
International Journal of Biomedical Imaging
Volume 2006, Article ID 12819, Pages 1–11
DOI 10.1155/IJBI/2006/12819

### Controlled Cardiac Computed Tomography

**Erwei Bai,[1] Chenglin Wang,[2] Ying Liu,[2] and Ge Wang[1]**

[1] *Department of Electrical and Computer Engineering and Department of Radiology, University of Iowa, Iowa City, IA 52242, USA*
[2] *Controlled Cardiac CT, LLC, Iowa City, IA, 52246, USA*

Received 3 December 2005; Accepted 5 March 2006

Recommended for Publication by Yibin Zheng

Cardiac computed tomography (CT) has been a hot topic for years because of the clinical importance of cardiac diseases and the rapid evolution of CT systems. In this paper, we propose a novel strategy for controlled cardiac CT that may effectively reduce image artifacts due to cardiac and respiratory motions. Our approach is radically different from existing ones and is based on controlling the X-ray source rotation velocity and powering status in reference to the cardiac motion. We theoretically show that by such a control-based intervention the data acquisition process can be optimized for cardiac CT in the cases of periodic and quasiperiodic cardiac motions. Specifically, we formulate the corresponding coordination/control schemes for either exact or approximate matches between the ideal and actual source positions, and report representative simulation results that support our analytic findings.

Copyright © 2006 Erwei Bai et al. This is an open access article distributed under the Creative Commons Attribution License, which permits unrestricted use, distribution, and reproduction in any medium, provided the original work is properly cited.

### 1. INTRODUCTION

Cardiovascular disease is the number one killer in the Western world. It is responsible for 1 of every 2.6 deaths in the United States. Cardiovascular disease was listed as a primary or contributing cause on about 1 408 000 death certificates annually. One in five persons has some form of cardiovascular diseases in the United States, account for 64 400 000 Americans (http://www.americanheart.org/). In 2004, the estimated cost of cardiovascular diseases is $368.4 billion. Needless to say, early detection of diseases is vitally important.

Electron-beam CT (EBCT) is a unique mode of medical X-ray CT, which is for early screening of coronary artery diseases [1–4]. EBCT is more accurate than relying on some traditional indicators, for example, high cholesterol levels, high blood pressure, obesity, diabetes, and so on, for determining an individual's risk for heart disease [5–10]. Specifically, EBCT can quantify calcification in the arteries, which is a sign of atherosclerosis. An EBCT-based coronary calcium score is, according to the American Heart Association (http://www.americanheart.org/), of significant predictive value of heart attacks or need for bypass or angioplasty over the next year or two. In addition, the American Heart Association has noted that EBCT is also useful in evaluating bypass graft potency, intra and congenital cardiac lesions, and quantifying ventricular muscle mass, chamber volumes, and systolic and diastolic functions. Hence, it has been recognized as the gold standard for detecting early heart diseases. In addition to its remarkable applications for dynamic anatomical imaging of cardiac structures, EBCT is also a powerful tool for physiological imaging [11]. However, there are three major limitations with the current EBCT techniques. First, it is not in cone-beam geometry, and hence it can only acquire a limited number of transverse slices through the heart. It has become clear that the multislice/cone-beam scanning is much more advantageous for a good portion or even the whole volume of the heart to be contained in a cone-beam, and reconstructed from a rapidly acquired data set for both high temporal resolution and excellent anatomical consistency. Second, the X-ray spot in EBCT is not sufficiently intensive to produce the image quality that the mechanical rotation-based scanners can achieve, which compromises contrast resolution in reconstructed images. Third, the EBCT scanner is both expensive and monstrous. As such, EBCT is far less accessible and less cost-effective than the main stream medical CT scanner that utilizes a mechanically rotated X-ray source.

Given the limitations of the EBCT, multislice/cone-beam CT has been growing into a strong competitor of the EBCT. Research on single-slice cardiac CT was initiated in [12, 13]. Multislice/cone-beam cardiac CT has been actively studied since then [14–21]. To obtain usable images of the cardiac or coronary features, a CT scan must be performed in coordination with an ECG signal or equivalent. There are two types of synchronization mechanisms: prospective and retrospective gating [22]. In the prospective mode, the heart is scanned at a pre-specified delay relative to a temporal landmark, such as after the peak of an R wave. Multiple prospectively triggered acquisitions can reveal different phases of the cardiac motion. In the retrospective mode, the heart is continuously scanned while the ECG is simultaneously recorded. Then, images are reconstructed from a retrospectively selected cardiac phase. Most of these cardiac imaging methods rely on retrospective gating to lock into consistent phases of the cardiac motion. An alternative to the ECG is referred to as the kymogram, which is based on an analysis on the trajectory of the center of the mass of the beating heart [18]. A knowledge-based cardiac CT algorithm was also developed [21]. Advantages of the cardiac phase-based approach clearly outperformed the classic half-scan methods. The manufacturers have now already switched from half-scan or partial-scan to multisector reconstruction algorithms for cardiac CT.

There are however two major problems with current gating-based cardiac CT methods [3, 23–25]. First, these existing methods are passive in their nature, and require that the cardiac motion and the X-ray source rotation must be at favorable relative frequencies; otherwise, the data sectors to be assembled for a complete data set would span a wide range of the cardiac status leading to a compromised image quality, or the data acquisition time would be too long to be practical (in a most unfavorable case, it could be impossible to acquire a complete data set) [17, 21]. Second, even in the favorable cases, retrospectively reconstructed cardiac images still suffer from substantial motion blurring because in practice each projection sector covers a projection angular range of a substantial length. Within such an angular range, the heart will move appreciably, especially when it is not in a relative stationary phase. As a benchmark, we routinely achieve 0.3 mm spatial resolution in spiral CT of the temporal bone where the motion magnitude is much less than that of the heart [21, 26]. On the other hand, the spatial resolution with cardiac CT is at best in the millimeter domain.

It is also important to comment that to understand etiology and pathogenesis of the human cardiovascular diseases as well as to develop prevention and treatment strategies, small animals have become common laboratory models. To use these animal models fully, the extension of cardiovascular imaging from patients to small animals, such as mice and rats, is imperative [27]. Because of the small size and high heart rate of a mouse, high spatial and temporal resolutions are required. Currently, an X-ray micro-CT scanner takes at least 20 seconds to acquire a full data set [27, 28]. Hence, cardiac micro-CT represents a daunting challenge. The recent small animal micro-CT work performed at Duke University showed the feasibility of respiratory and cardiac-gated micro-CT [29], but their system runs very slowly (not well suited for high throughput [29]), and requires that the mouse is placed vertically and rotated asynchronously, violating the natural physiological conditions.

All the above problems demonstrate themselves as motion-induced image artifacts. To this end, we propose a new approach, which is radically different from any existing technique. Our primary insight is that cardiac imaging can be optimally performed by integrating modern control and imaging techniques, that is, by adaptively controlling the imaging device according to the real-time analysis and prediction of an individualized respiratory and cardiac motion functions, little artifacts would be left. It is expected that the spatial resolution with the proposed controlled cardiac CT should be significantly better than that with current cardiac CT, and eventually made to be comparable to that with temporal bone CT.

Two questions come to the mind immediately. Is controlling source rotation velocity of a CT a realistic task and how to coordinate prediction and control? This paper discusses the prediction and coordination questions, and sets the theoretical foundation for controlled cardiac CT/micro-CT. We only make a comment on the control hardware. Control technology is a matured area and a number of advanced control methods, including robust, adaptive, nonlinear, fuzzy, and intelligent controls, have been proposed, theoretically justified and practically tested. One can easily find a precision control including hardware and software everywhere in every day life from airplanes, electronics to microsurgery. A CT with controllable source rotation velocity is completely feasible which will be demonstrated in the paper.

### 2. PROBLEM STATEMENTS

To describe coordination of prediction and control in a precise term, we define a few terminologies.

(i) Let $v(t)$ be the volume or phase of the object, for example, a heart, to be scanned at time $t$. It is a function of time and could be periodic or nonperiodic.

(ii) Let $a_l = (l-1)/p \cdot 2\pi$, $l = 1, 2, \ldots, p$, be $p$ evenly spaced angles. Evenly spaced is for simplicity only. Non-evenly spaced angles can be considered similarly.

(iii) Let $s(t)$ represent the angle, at time $t$, of the CT X-ray source. It is a function of time and its rotational velocity is assumed to be controllable.

In this paper, we assume that at any given time, only one projection can be taken. The results can be easily extended to multiple source/sensor cases.

First consider a simple case of a periodic $v(t)$ with period $P$, that is,

$$v(t) = v(t + P) \quad \forall t. \tag{1}$$

Define the volume levels of the object

$$\begin{gathered} v(t_i), \quad i = 1, 2, \ldots, q, \ v(t_i) \neq v(t_j), \\ 0 \leq t_1 \leq t_2 \leq \cdots \leq t_q < P, \end{gathered} \tag{2}$$

and the corresponding two sequences

$$\begin{aligned} L_i &= \{v(t_i), v(t_i + P), \ldots, v(t_i + (p-1)P), \ldots\}, \\ T_i &= \{t_i, t_i + P, t_i + 2P, \ldots, t_i + (p-1)P, \ldots\}. \end{aligned} \tag{3}$$

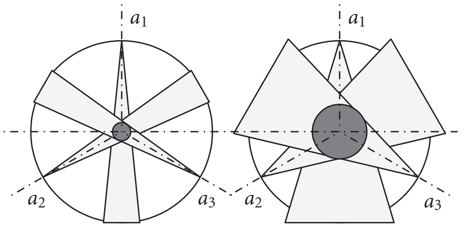

FIGURE 1: Angles and levels.

Here, it is recognized that for the same cardiac volume level, the structure of the heart could be different in the expanding and contracting phases. Obviously, $v(t) = v(t_i)$ if $t \in T_i$. Volume levels are the levels where the object is required to be imaged. Figure 1 shows the interaction of $v(t_i)$'s and $a_l$'s for $q = 2$ and $p = 3$.

Now, for the prescribed angles $a_l$'s and the levels $v(t_i)$'s, solving the problem of CT imaging without motion interference is equivalent to solving the following problem.

*Problem 1.* (a) Find a source angle profile $s(t)$ as a function of time $t$ so that for every angle $a_l$, $l = 1, \dots, p$, at every level $i$, $i = 1, \dots, q$, there exists a time $t$ such that

$$s(t) = a_l, \qquad v(t) \in L_i. \tag{4}$$

In other words, the source angle is $a_l$ when the volume level is at $v(t_i)$ for all $i, l$. In short, the source covers all the angles $a_l$, $l = 1, \dots, p$, exactly at all the levels $v(t_i)$, $i = 1, \dots, q$.

(b) Among all the solutions $s(t)$'s, find one that requires the minimum time to cover all the angles for all the levels.

In general, a constant source rotation velocity $s(t) = \alpha t$, for some constant $\alpha$, does not solve Problem 1.

### 3. SOURCE ROTATION CONTROL FOR EXACT MATCH

### 3.1. Periodic $v(t)$

To solve Problem 1, we now consider controlled source rotation velocity $s(t)$. Initially, intuition seems to suggest that a solution of Problem 1 would need a very fast and complicated profile of $s(t)$. This is however not the case. To this end, we make two observations.

(i) At each level $i$, $p$ projections at $p$ different angles $a_l, l = 1, \dots, p$, have to be taken.

(ii) Only times that projections can be taken exactly at the level $i$ are

$$t \in L_i = \{t_i, t_i + P, \dots, t_i + (p-1)P, \dots\}. \tag{5}$$

Because of $p$ angles at each level and the assumption that at any given time, only one projection can be made, the minimum time needed for the level $i$ is $t_i + (p-1)P$. This leads to the following lemma.

**Lemma 1.** *The minimum time needed to cover all the angles at all the levels for any solution $s(t)$ is at least*

$$\max_i \left(t_i + (p-1)P\right) = t_q + (p-1)P. \tag{6}$$

This is a lower bound on the minimum time. Any solution $s(t)$ of Problem 1(a) achieving the lower bound (6) solves Problem 1(b). Base on these observations, we have the following

**Theorem 1.** *Up to a possible permutation on $a_l$, a necessary and sufficient condition for $s(t)$ to be a solution of Problem 1(b) is*

$$s(t_i + (l-1)P) = a_l, \quad i = 1, \dots, q, \ l = 1, \dots, p. \tag{7}$$

This is an interpolation problem and many solutions, for example, polynomial or spline interpolations, exist. To compare the results with the gating method for a constant source rotation velocity $s(t)$, we give a numerical example. First note the gating method is to specify the X-ray source rotation velocity and data acquisition timing based on the cardiac rate so that data acquisition timing is at the consistent heart volume levels while the X-ray source is distributed in an appropriate angular range. Now, let the heart volume be

$$v(t) = r_1 + r_2 \cos(2\pi t) \tag{8}$$

for some constants $r_1$, $r_2$. $v(t)$ is periodic with period $P = 1$ (sec). Suppose the level that we are interested in is at $t_1 = 5/8$ or

$$v(t_1) = r_1 + r_2 \cos\left(2\pi\left(\frac{5}{8} + m\right)\right). \tag{9}$$

Since $v(t)$ is periodic, $v(t_1 + m) = v(t_1)$ for any integer $m$. Now, without loss of generality, let the angles be $a_l = 2\pi(l-1)/3$, $l = 1, 2, 3$. To have exact angles $a_l = 2\pi(l-1)/3$ at the level $v(t_1)$, we must have for some $m$'s,

$$s(t_1 + m) = \alpha\left(\frac{5}{8} + m\right) = \frac{2\pi(l-1)}{3}, \quad \text{mod } 2\pi, \ l = 1, 2, 3. \tag{10}$$

It can be shown that there does not exist a constant source rotation velocity $s(t) = \alpha t$, so the above three equations can be satisfied simultaneously. Approximate solutions do exist. To quantify the error, we define the minimum error between the given angles and all possible X-ray source angles at the given heart volume level $v(t_1)$ as

$$e = \min_{\alpha > 0} \min_{m \geq 0} \max_l \left\{\alpha\left(\frac{5}{8} + m\right) - \frac{2\pi(l-1)}{3}\right\}, \quad \text{mod } 2\pi. \tag{11}$$

Table 1, converted to degrees, shows $e$ and the corresponding optimal $\alpha^*$ and $m^*$. For instance, if the error is required to be no larger than 0.631°, the best $\alpha \in (0, \infty)$ is $0.316\pi$ that results in the minimum error 0.631° at time $t = 82+5/8$ (sec).

TABLE 1: Minimum errors and the corresponding times.

| | | | |
|---|---|---|---|
| $\alpha^*$ | $0.316\pi$ | $0.036\pi$ | $0.042\pi$ |
| $e$ | $0.631°$ | $0.45°$ | $0.045°$ |
| $m^* + 5/8$ | $82 + 5/8$ | $172 + 5/8$ | $433 + 5/8$ |

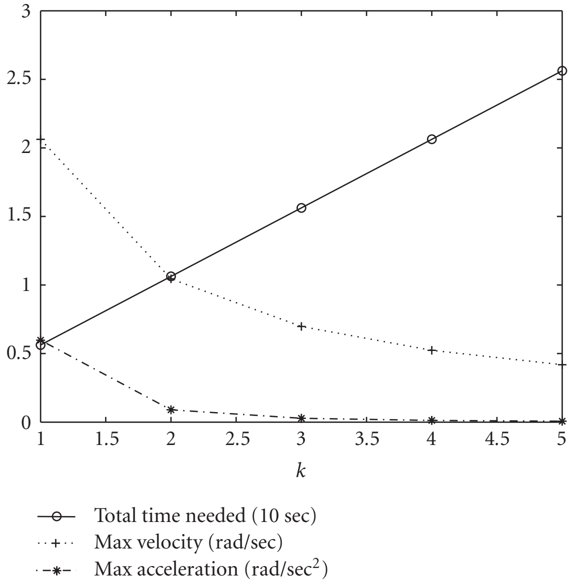

FIGURE 2: Time needed versus max velocity/acceleration.

Clearly, the more accurate the solution is required, the longer time the scan has to take.

Now, let the X-ray source velocity be controllable as defined by

$$s(t) = \begin{cases} at + bt^2 + ct^3, & 0 \le t \le \dfrac{5}{8} + 3k, \\ \dfrac{2\pi}{3k}\left(t - \dfrac{5}{8} - 3k\right), & t \ge \dfrac{5}{8} + 3k. \end{cases} \tag{12}$$

with

$$\begin{pmatrix} 0 & 2 & 6\left(\frac{5}{8}+3k\right) \\ 1 & 2\left(\frac{5}{8}+3k\right) & 3\left(\frac{5}{8}+3k\right)^2 \\ \left(\frac{5}{8}+3k\right) & \left(\frac{5}{8}+3k\right)^2 & \left(\frac{5}{8}+3k\right)^3 \end{pmatrix} \begin{pmatrix} a \\ b \\ c \end{pmatrix} = \begin{pmatrix} 0 \\ \frac{2\pi}{3k} \\ 2\pi \end{pmatrix}. \tag{13}$$

It is easily verified that this X-ray source $s(t)$ covers three angles exactly at the required volume level $v(5/8 + m)$ for any integer $k > 0$, that is, $s(5/8 + 3k) = 0(+2\pi)$, $s(5/8 + 4k) = 2 * pi/3(+2\pi)$, and $s(5/8 + 5k) = 4 * \pi/3(+2\pi)$. $k$ balances the time required and the maximum velocity and acceleration. A small $k$ gives rise to a small maximum velocity and acceleration but at the same time a longer time to cover three angles. A large $k$ results in a short time with a price of a large maximum velocity and acceleration. Figure 2 shows the relationship between the time needed to cover three angles exactly and the corresponding maximum velocity and acceleration as a function of $k$. It is important to note that for a variable source rotation velocity $s(t)$ to work, the maximum velocity and acceleration do not have to be large. Whether or not there is control of the source rotation velocity of $s(t)$ is vital.

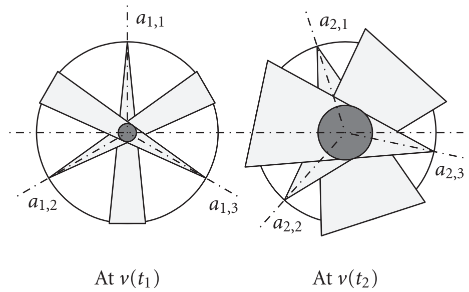

FIGURE 3: Angles and levels for Problem 2.

If the angle accuracy is within 0.05°, the time required as shown in Table 1 is $(433 + 5/8)$ (sec) for the best constant source rotation velocity $s(t)$. By using a variable source rotation velocity $s(t)$ as defined above, say $k = 5$, it takes $(25+5/8)$ (sec) with the maximum velocity 0.42 (rad/sec) and the maximum acceleration 0.0064 (rad/sec$^2$). This kind of velocity and acceleration imposes no problem to hardware at all for most commercially available servo systems. For instance, suppose the X-ray source and sensor weigh 100 kg and the gantry diameter is 1m, an acceleration of 0.0064 (rad/sec$^2$) results in a torque 0.16 Nm. The Panasonic servo motor series A, E, and S and other commercially available products can easily meet this kind of requirements.

Clearly, a constant source rotation velocity $s(t)$ is not a solution and this adds complexity to the control of $s(t)$. Thus, it is interesting to note that the following modified Problem 2 allows an constant but not prefixed velocity solution.

*Problem 2.* (a) Find a source angle profile $s(t)$ as a function of time $t$ so that for every $i$ at every $l$, there exists a corresponding time $t$ such that

$$s(t) = a_{i,l} = a_l + \theta_i, \quad v(t) \in L_i, \tag{14}$$

where $\theta_i$ is independent of $l$. The interpretation is that at any level $i$, projections have to be taken at $p$ evenly spaced angles $a_{i,l} = a_l + \theta_i = ((l-1)/p)2\pi + \theta_i, l = 1, \dots, p$, with the same offset angle $\theta_i$. However, the offset angle $\theta_i$ at level $i$ may not be the same as the offset angle $\theta_j$ at level $j$.

(b) Among all the solutions $s(t)$'s, find one that requires the minimum time.

The difference between Problems 1 and 2 is that Problem 1 requires that at any level $v(t_i)$, samples are taken exactly at $p$ evenly spaced angles $a_l$'s while Problem 2 does not require that $p$ evenly spaced angles at level $i$ are the same as that at level $j$, as illustrated in Figure 3. This makes sense for practical applications where an accurate image of level $v(t_i)$

is reconstructed as long as $p$ is large enough independent of whether the same angles are used for level $j \neq i$. Surprisingly, the seeming complicated Problem 2 is solved by a constant source rotation velocity $s(t)$.

**Theorem 2.** (1) *Let $m \geq 0$ and $k > 0$ be any integers and consider*

$$s(t) = \left(\frac{2\pi}{p} + 2m\pi\right)\frac{t}{Pk} = \frac{2\pi}{(p/(1+mp))Pk}t = \frac{2\pi}{P_c}t, \tag{15}$$

*where $P_c = (p/(1+mp))Pk$ is the period of $s(t)$. Then,*

$$t = t_i + (l-1)Pk \Longrightarrow s(t) = a_{i,l} = a_l + \theta_i. \tag{16}$$

(2) *The X-ray source $s(t)$ defined above with $k = 1$ solves Problem 2. In other words, $s(t)$ covers the angles $a_{i,l}$'s exactly at precise volume levels $v(t_i)$ with the minimum time.*

*Proof.* Let $i \in [1,\ldots,q]$ and $l \in [1,\ldots,p]$. At time $t = t_i + (l-1)Pk$,

$$\begin{aligned} s(t) &= \left(\frac{2\pi}{p} + 2m\pi\right)\left(\frac{t_i}{(Pk)} + (l-1)\right) \\ &= \frac{l-1}{p}2\pi + \underbrace{\left(\frac{2\pi}{p} + 2m\pi\right)\frac{t_i}{(Pk)}}_{\theta_i} + 2\pi m(l-1) \\ &= a_{i,l} = a_l + \theta_i \end{aligned} \tag{17}$$

because $2\pi m(l-1)$ is an integer multiple of $2\pi$. Thus, $s(t)$ covers all the angles $a_{i,l}$ at all the levels $i$. Moreover, with $k = 1$, it covers all the angles at all the levels with the minimum time $t_q + (p-1)P$. This completes the proof. □

*Remark 1.* (1) The reasons why the X-ray source rotation $s(t)$ of (15) can cover all the angles for all the levels are the following. (a) It is constant but selected by the user depending on the period of the heart volume $v(t)$ and not refixed. (b) Different offset angles $\theta_i$'s are allowed for different levels. This is key difference between $s(t)$ of (15) and methods currently available in the literature.

(2) Clearly, the minimum time is achieved with the minimum $k$. On the other hand, the small values of $k$ imply high velocities of $s(t)$. There is a tradeoff between the minimum time and the maximum velocity. The balance can be achieved by adjusting $k$. Note that the velocity of $s(t)$ is $2\pi/(p/(1+mp))Pk$ and moreover the acceleration of $s(t) = (2\pi/P_c)t$ is always zero for any $m, k$.

(3) If the imaging device has multiple sources/detectors, say $n$ sources/detectors, the minimum time could be reduced by a factor of $n$.

(4) The optimal $s(t)$ does not have to be fast. For instance, let $m = 0$ and $p = 360$, that is, there are 360 evenly spaced angles. Then,

$$P_c = 360\,\text{kP}. \tag{18}$$

The CT rotates 360 k times slower than the motion of the object. In general, $P_c = p\,\text{kP}$ if $m = 0$. For instance, a heart rate is 60 cycles per minute and the CT could rotate as slow as 1 cycle in every 6 minutes when $k = 1$ and even slower when $k > 1$.

(5) Though $s(t)$ does not have to be fast, it has to be accurate. For instance, let $k = 1$ and write a constant source rotation velocity $s(t)$ as, for some $d$,

$$s(t) = \left(d\frac{2\pi}{p} + 2m\pi\right)\frac{t}{Pk}. \tag{19}$$

At $k = 1$ and $t = t_i + (l-1)P$,

$$\begin{aligned} s(t) &= \left(d\frac{2\pi}{p} + 2m\pi\right)\left(\frac{t_i}{P} + (l-1)\right) \\ &= d \cdot \frac{l-1}{p}2\pi + \underbrace{2m\pi t_i/P}_{\theta_i} + 2m\pi(l-1) \\ &= d \cdot \frac{l-1}{p}2\pi + \theta_i, \end{aligned} \tag{20}$$

$d$ determines the increment. If $s(t)$ is not exactly a constant

$$d = \bar{d} + \Delta d(t), \tag{21}$$

where $\Delta d(t)$ indicates variation of the velocity in $s(t)$, then, at time $t = t_i + (l-1)P$,

$$\begin{aligned} s(t) &= \left(d\frac{2\pi}{p} + 2m\pi\right)\left(\frac{t_i}{P} + (l-1)\right) \\ &= \bar{d} \cdot \frac{l-1}{p}2\pi + \theta_i + \underbrace{\Delta d(t_i + (l-1)P) \cdot \frac{l-1}{p}2\pi}_{\text{error caused by } \Delta d}. \end{aligned} \tag{22}$$

Obviously, the last term controls the accuracy.

(6) The maximum difference between $\theta_i$ and $\theta_j$ for any $k$ and $m$ is

$$\max_{i,j} |\theta_i - \theta_j| \leq \frac{360}{2p}. \tag{23}$$

Now, consider the example (8) again. Suppose three heart volume leaves $v(t_1) = v(0)$, $v(t_2) = v(1/2)$, and $v(t_3) = v(5/8)$ are interested and at each level, three angles $a_l = (l-1)2\pi/3, l = 1,2,3$, are required. We can simply set $s(t) = (2\pi/P_c)t$. Since $P = 1$ and $p = 3$, with $m = 0$ and $k = 1$, we have $s(t) = (2\pi/3)t$ and

$$\theta_1 = 0, \qquad \theta_2 = 1.0472, \qquad \theta_3 = 1.3090 \tag{24}$$

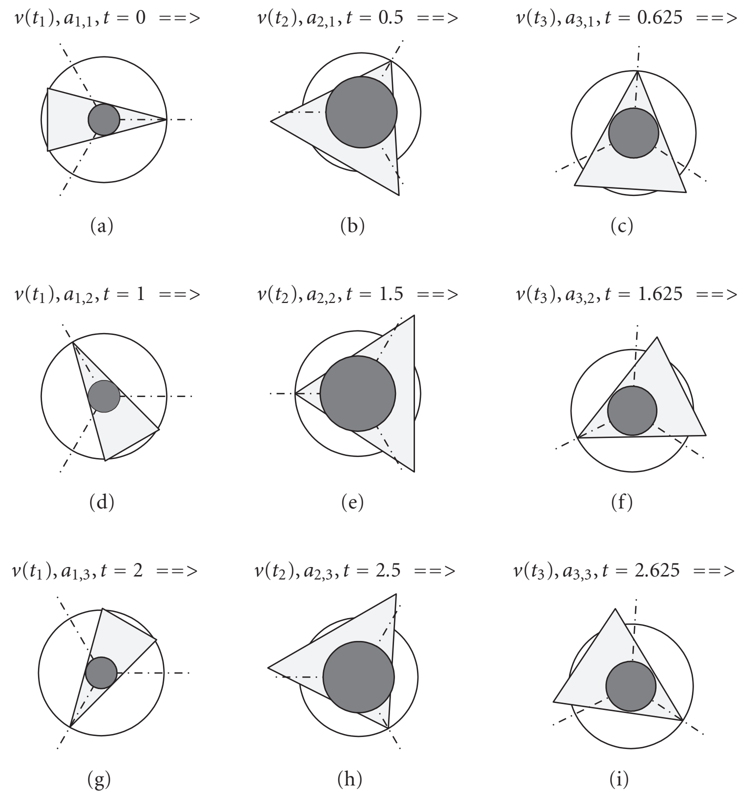

Figure 4: The sequence that s(t) covers $a_{i,l}$.

that result in

$$a_{1,l} = \frac{(l-1)2\pi}{3} + \theta_1,$$

$$a_{2,l} = \frac{(l-1)2\pi}{3} + \theta_2, \tag{25}$$

$$a_{3,l} = \frac{(l-1)2\pi}{3} + \theta_3.$$

This $s(t)$ covers three angles exactly at precise levels as shown in Figure 4. Also, note that the heart volume period is 1 (sec) and the X-ray source rotation period is 3 (sec), three times slower than the heart rate but still able to cover everything exactly with the minimum time.

Theorems 1 and 2 show that artifacts can be completely eliminated if the motion is periodic and the source rotation velocity of $s(t)$ is controllable. For a periodic motion, determination of the period is trivial. It can be estimated and predicted based on the analysis of the first a few cycles of ECG signals.

### 3.2. *Quasiperiodic* $v(t)$

To deal with a nonperiodic $v(t)$, let us recall the properties of a periodic function. A function $v(t)$ is periodic with period $P$ if and only if it is completely determined by the first period $t \in [0, P)$ and $v(t), t \in [(l-1)P, lP)$, is a translated version of $v(t)$, $t \in [0, P)$. Mathematically, for $t \in [(l-1)P, lP)$, $l = 1, 2, \dots,$

$$v(t)\big|_{t\in[(l-1)P,lP)} \doteq v(t-(l-1)P)\big|_{t-(l-1)P\in[0,P)}. \tag{26}$$

In CT imaging, we assume that the dominant motion is cardiac and respiratory movement. Then, we observe that such motion can be nonperiodic, that is, the time that takes to finish one cycle may be different from the time to finish another cycle. Independent of time, the cycle however always starts at the minimum volume level extending to the maximum volume level, and then ends at the minimum volume level. Such a motion can be characterized by a class of functions that we call quasiperiodic. Let

$$0 = P_0 < P_1 < P_2 < \cdots P_l < \cdots \tag{27}$$

be a monotone increasing sequence. The interval $P_{l+1} - P_l$ is the $l$th "period" of $v(t)$, that is, the time $v(t)$ finishes a cycle from the minimum volume to the maximum volume and then back to the minimum volume level. In general,

$$P_{l+1} - P_l \neq P_l - P_{l-1}, \tag{28}$$

$v(t)$ may be completely determined by its first "period" $v(t) = v_1(t), t \in [0, P_1)$. More precisely, let

$$\alpha_l = \frac{P_l - P_{l-1}}{P_1 - P_0}. \tag{29}$$

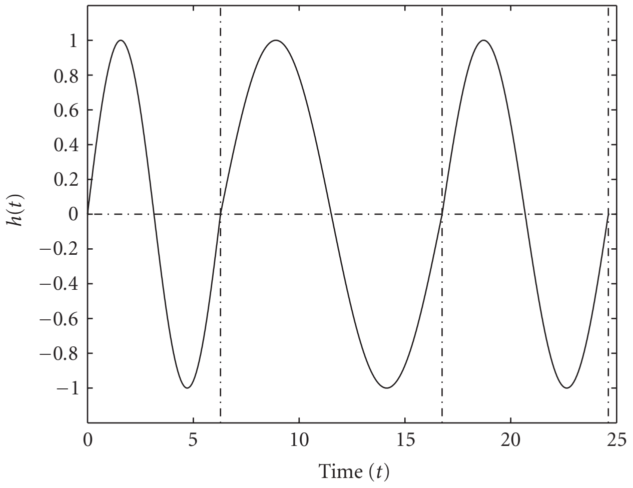

FIGURE 5: A quasiperiodic function.

Then, for $t \in [(l-1)P, lP)$,

$$v(t)\big|_{t\in[(l-1)P,lP)} \doteq v_1\left(\frac{P_1-P_0}{P_l-P_{l-1}}(t-P_{l-1})\right) = v_1\left(\frac{1}{\alpha_l}(t-P_{l-1})\right)\Big|_{(1/\alpha_l)(t-P_{l-1})\in[0,P_1)}. \quad (30)$$

The intuition is that $v(t)$ is periodic. However, in each "period" $[P_{l-1}, P_l)$, $v(t)$ is an expanded or compressed version of the first "period" $v_1(t), t \in [0, P_1)$. We give an example as shown in Figure 5:

$$v(t) = \begin{cases} \sin(t), & 0 \le t < 2\pi, \\ \sin(0.6(t-2\pi)), & 2\pi \le t < 2\pi + \dfrac{2\pi}{0.6}, \\ \sin\left(0.8\left(t - 2\pi - \dfrac{2\pi}{0.6}\right)\right), & 2\pi + \dfrac{2\pi}{0.6} \le t < 2\pi, \\ & +\dfrac{2\pi}{0.6} + \dfrac{2\pi}{0.9} \\ \vdots & \vdots \end{cases} \quad (31)$$

Because $v(t)$ is no longer periodic, the definition of the volume level has to be modified. For the first period $v(t) = v_1(t), t \in [0, P_1)$, the levels are well defined:

$$v_1(t_i), \quad i = 1,\ldots,q,\ 0 \le t_1 < t_2 < \cdots < t_q < P_1, \quad v_1(t_i) \ne v_1(t_j). \quad (32)$$

For the $l$th "period," we note $P_{l-1} \le \alpha_l t_i + P_{l-1} < P_l$ and at $t = \alpha_l t_i + P_{l-1}$,

$$v(t) = v_1\left(\frac{1}{\alpha_l}(t-P_{l-1})\right) = v_1(t_i), \quad i = 1,\ldots,q. \quad (33)$$

Thus, the $i$th volume level can be defined as

$$L_i = \{v(\alpha_1 t_i + P_0), v(\alpha_2 t_i + P_1), \ldots, v(\alpha_p t_i + P_{p-1}) \cdots\} = \{v(t_i), v(\alpha_2 t_i + P_1), \ldots, v(\alpha_p t_i + P_{p-1}) \cdots\} \quad (34)$$

or in time domain

$$T_i = \{\alpha_1 t_i + P_0, \alpha_2 t_i + P_1, \ldots, \alpha_p t_i + P_{p-1} \cdots\} = \{t_i, \alpha_2 t_i + P_1, \ldots, \alpha_p t_i + P_{p-1} \cdots\}. \quad (35)$$

*Problem 3* (for quasiperiodic $v(t)$'s). (a) Find a source angle profile $s(t)$ as a function of time $t$ that

$$s(\alpha_l t_i + P_{l-1}) = a_{i,l}, i = 1,\ldots,q, l = 1,\ldots,p, \quad (36)$$

where $a_{i,l}$ was defined before.

(b) Among all the solutions $s(t)$'s, find one that requires the minimum time.

**Theorem 3.** *For each* $[P_{l-1}, P_l)$, *let*

$$s(t) = \beta_{0l} + \beta_{1l}\left[\frac{1}{\alpha_l}(t-P_{l-1})\right] + \beta_{2l}\left[\frac{1}{\alpha_l}(t-P_{l-1})\right]^2 + \cdots + \beta_{ql}\left[\frac{1}{\alpha_l}(t-P_{l-1})\right]^q, \quad t \in [P_{l-1}, P_l), \quad (37)$$

*where*

$$\beta_{0l} + \beta_{1l} t_i + \cdots + \beta_{ql} t_i^q = a_{i,l}, \quad l = 1,\ldots,p,\ i = 1,\ldots,q, \quad (38)$$

*or equivalently*

$$\begin{pmatrix} 1 & t_1 & \cdots & t_1^q \\ \vdots & \vdots & \ddots & \vdots \\ 1 & t_q & \cdots & t_q^q \end{pmatrix} \begin{pmatrix} \beta_{0l} \\ \vdots \\ \beta_{ql} \end{pmatrix} = \begin{pmatrix} a_{1,l} \\ \vdots \\ a_{q,l} \end{pmatrix}, \quad l = 1,\ldots,p. \quad (39)$$

*Then,* $s(t)$ *of* (37) *solves Problem 3(b).*

*Proof.* Again, at $t = \alpha_l t_i + P_{l-1}$,

$$v(t) = v_1\left(\frac{1}{\alpha_l}(t-P_{l-1})\right) = v_1(t_i), \quad i = 1,\ldots,q,$$

$$s(t) = \beta_{0l} + \beta_{1l} t_i + \cdots + \cdots + \beta_{ql} t_i^q = a_{i,l}, i = 1,\ldots,q, \quad l = 1,\ldots,p. \quad (40)$$

Therefore, $s(t)$ of (37) covers all the angles $a_{i,l}$ at all the levels $v(\alpha_l t_i + P_{l-1})$. The proof of the minimum time is similar as before. □

*Remark 2.* (1) Unlike the periodic case, any constant $s(t)$ fails to cover all angles $a_{i,l}$'s at all levels with the minimum time, even with slack variables $\theta_i$'s added.

(2) The idea of $s(t)$ in (37) is again to interpolate $a_{i,l}$ at $t = \alpha_l t_i + P_{l-1}$. Thus, the solution of $s(t)$ is not unique and any other form of $s(t)$ that interpolates $a_{i,l}$ at $t = \alpha_l t_i + P_{l-1}$ is also a solution.

(3) The source rotation velocity of $s(t)$ in $t \in [P_l, P_{l-1})$ is

$$\begin{aligned}\max |s'(t)| &= \left| \frac{\beta_{1l}}{\alpha_l} + 2\frac{\beta_{2l}}{\alpha_l}\left(\frac{(P_l - P_{l-1})}{\alpha_l}\right) + \cdots + \right. \\ &\quad \left. + q\frac{\beta_{ql}}{\alpha_l}\left(\frac{(P_l - P_{l-1})}{\alpha_l}\right)^{q-1} \right| \\ &\le \left|\frac{\beta_{1l}}{\alpha_l}\right| + 2\left|\frac{\beta_{2l}}{\alpha_l}\right|(P_1 - P_0) + \cdots + \\ &\quad + q\left|\frac{\beta_{ql}}{\alpha_l}\right|(P_1 - P_0)^{q-1}.\end{aligned} \tag{41}$$

In general if the "frequency" of $v(t)$ is high, that is, the "period" $P_{l+1} - P_l$ is small. This implies that the velocity $s'(t)$ is small, provided that $|\beta_{1l}/\alpha_l|$ is not large. In the case that the maximum velocity $|s'(t)|$ is of concern, interpolation can be done for both $s(\alpha_l t_i + P_{l-1})$ and the constraints on $|s'(\alpha_l t_i + P_{l-1})|$. There exists a large volume of works in the literature in this direction, referred to as Hermite- or Fejer-type interpolations.

(4) $s(t)$ of Problem 3 may have discontinuities at $P_l$'s. If these discontinuities are of concern, spline interpolations can be used. The result is a smooth piecewise polynomial at a price of increased computational complexity.

Control Problem 3 is solved in Theorem 3 for quasiperiodic $v(t)$'s provided the period $P_l - P_{l-1}$ is available. Estimation of the period $P_l - P_{l-1}$ is rather straightforward for quasiperiodic functions. $v(t)$ is quasiperiodic, so the form of $v(t)$ can be determined from its first a few cycles. Then, in theory, the period can be calculated by observing some points of $v(t)$. To be precise, take $v(t)$ of (31) as an example. Suppose the form of $v(t)$ has been determined from its first cycle and we want to find the period $P_2 - P_1$. Note $v(t) = \sin(c(t - 2\pi))$, $t \in (P_1, P_2)$ for some unknown constant $c$. If $c$ is determined, the period $P_2 - P_1 = 2\pi/c$. To estimate $c$, what we need is one observation $v(\bar{t})$, $\bar{t} \in (P_1, P_2)$, which leads to

$$v(\bar{t}) = \sin(c(\bar{t} - 2\pi)) \Longrightarrow c = \frac{1}{\bar{t} - 2\pi}\arg\sin(v(\bar{t})). \tag{42}$$

In implementation, because of noises, it is preferred to have several observations and the estimate $c$ takes the form of the average of individual estimates.

To illustrate the idea, we consider an example:

$$v(t) = \begin{cases} 1 - 0.5\cos(2 * \pi * t), & t \in [0, 1), \\ 1 - 0.5\cos\left(2 * \pi * \dfrac{t}{0.8}\right), & t \in [1, 1.8), \\ 1 - 0.5\cos\left(2 * \pi * \dfrac{t}{0.5}\right), & t \in [1.8, 2.3). \end{cases} \tag{43}$$

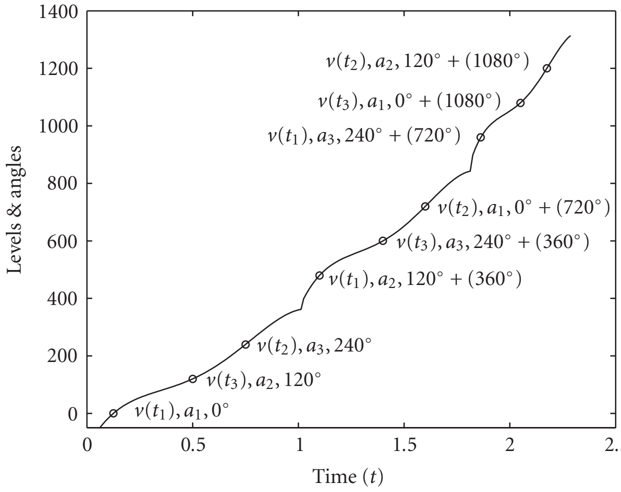

Figure 6: Controlled X-ray source for a quasiperiodic function.

This is no longer periodic but quasiperiodic. Suppose three volume levels $v(t) = 0.64, 1, 1.5$ and three angles $a_l = (l - 1) * 2 * \pi/3, l = 1, 2, 3$, are interested. Figure 6 shows the computer simulation results based on the polynomial interpolation and the estimates of the unknown periods. Clearly, three angles at precise three volume levels are imaged.

The advantage of the approach is that no other signal is needed. If other signals, for example, ECG signals, are available, the period can also be calculated from the gating of ECG signals.

### 4. SOURCE ROTATION CONTROL FOR NONEXACT MATCH

In practice, the minimum time to cover all the angles at all the levels can be important. In the previous discussions, the exact level $v(t_i)$ and exact angle $a_l$ have to be matched to take images. This is referred to as the exact match and in this situation, the minimum time needed for any solution $s(t)$ to cover all the angles at all the levels is $\alpha_p t_q + P_{p-1}$. From a practical point of view, the exact match is not necessary as long as errors are small enough. We discuss the nonexact match in both angle and level. By nonexact matches, we mean that, at each level $i$, images do not have to be taken exactly at $v(\alpha_l t_i + P_{l-1})$ but in a small neighborhood of $v(\alpha_l t_i + P_{l-1})$. Similarly, angle $a_l$ does not have to be exact but within a small neighborhood.

To this end, let, at level $i$, the error bound $\delta > 0$ and the corresponding times $\underline{t}_{i,l} \le t_i \le \bar{t}_{i,l}$ be given that satisfy

$$\max_{t \in [\alpha_l \underline{t}_{i,l} + P_{l-1}, \alpha_l \bar{t}_{i,l} + P_{l-1}]} |v(t) - v(\alpha_l t_i + P_{l-1})| \le \delta. \tag{44}$$

Further define

$$\begin{aligned}\Delta_{i,l} &= \left[\min_{t \in [\alpha_l \underline{t}_{i,l} + P_{l-1}, \alpha_l \bar{t}_{i,l} + P_{l-1}]} v(t), \max_{t \in [\alpha_l \underline{t}_{i,l} + P_{l-1}, \alpha_l \bar{t}_{i,l} + P_{l-1}]} v(t)\right], \\ \tau_{i,l} &= [\alpha_l \underline{t}_{i,l} + P_{l-1}, \alpha_l \bar{t}_{i,l} + P_{l-1}].\end{aligned} \tag{45}$$

The neighborhood of $v(\alpha_l t_i + P_{l-1})$ is then defined as $\Delta_{i,l}$ with the corresponding time interval $\tau_{i,l}$. Clearly,

$$v(\alpha_l t_i + P_{l-1}) \in \Delta_{i,l}, \qquad \max_{t \in \tau_{i,l}} |v(t) - v(\alpha_l t_i + P_{l-1})| \leq \delta, \tag{46}$$

and $\tau_{i,l}$ is the time interval in which the images for any angle $a_l$, $l = 1, \dots, p$, can be taken at the level $i$. By the same token, the volume level $i$ in a nonexact match case can be defined as

$$L_i = \{\Delta_{i,1}, \Delta_{i,2}, \dots, \Delta_{i,p}, \dots\} \tag{47}$$

or in time domain

$$T_i = \{\tau_{i,1}, \tau_{i,2}, \dots, \tau_{i,p}, \dots\}. \tag{48}$$

In the nonexact match case, elements of $L_i$ and $T_i$ and intervals not points. For simplicity, we assume $\tau_{i,l}$ and $\tau_{j,k}$ do not overlap for $i \neq j$ and/or $l \neq k$.

Now, the problem can be modified as to find a source angle profile $s(t)$ with the minimum time satisfying

$$\begin{gathered} |s(t) - a_l| \leq \epsilon, \quad l = 1, \dots, p, \\ t \in \tau_{i,k}, \quad \text{for some } k, \end{gathered} \tag{49}$$

where $\epsilon > 0$ is the given angle error bound. If the velocity and acceleration of $s(t)$ are of concern, the constraints $|s'(t)| \leq M_1, |s''(t)| \leq M_2$ can be added in optimization, where $M_1$ and $M_2$ reflect the constraints of the physically achievable velocity and acceleration of the source angle $s(t)$.

### 5. CONCLUDING REMARKS

Our primary insight is that cardiac imaging can be optimally performed by integrating modern control and imaging techniques, that is, by adaptively controlling the imaging device according to the real-time analysis and prediction of an individualized respiratory and cardiac motion functions. The work intends to synchronize the X-ray source rotation and cardiac CT for unprecedented performance. Clinically, our work may induce significant advancement of the field of cardiology.

Although the studies have been focused on the cardiac motion, our methodology can be in principles applied to compensate for either the respiratory motion effect or the combined effect due to both cardiac and respiratory motions. Also, with the controlled data acquisition process the X-ray source positions will be distributed quite randomly along the scanning circle. Therefore, new CT image reconstruction methods should be developed for optimal image quality in this context. Some Feldkamp-type/Katsevich-type algorithms can be developed for that purpose, similarly to what reported in the literature [30–34]. Further efforts are needed to formulate such integrated control strategies and associated image reconstruction methods, and evaluate their performance systematically.

It should be underlined that there is no difficulty in implementation of the proposed control schemes in terms of hardware limitations. The hardware requirements can be easily met by commercially available products, for instance, the Panasonic servo motor and driver series. The fact is that the control theory and techniques have been well developed over the past decades, but the problem or the opportunity is that control and imaging techniques have never been combined before for cardiac CT.

In conclusion, we have proposed an innovative idea and specific schemes to eliminate CT image artifacts due to cardiac and respiratory motions, and demonstrated the feasibility and importance of controlled cardiac CT. We feel that the idea reported here may open a new frontier of biomedical imaging. We will continue to work along this direction.

### REFERENCES

[1] D. P. Boyd and M. J. Lipton, “Cardiac computed tomography,” *Proceedings of the IEEE*, vol. 71, pp. 198–307, 1983.

[2] Y. Liu, H. Liu, Y. Wang, et al., “Half-scan cone-beam CT fluoroscopy with multiple x-ray sources,” *Medical Physics*, vol. 28, no. 7, pp. 1466–1471, 2002.

[3] E. Mousseaux, A. Hernigou, M. Auguste, et al., “Dynamic cardiovascular studies with electron beam computed tomography,” *Journal de Radiologie*, vol. 75, no. 12, pp. 669–674, 1994.

[4] B. H. Thompson and W. Stanford, “Electron beam computed tomographic imaging of aortic aneurysms and dissections,” *The Journal of Invasive Cardiology*, vol. 6, no. 6, pp. 213–227, 1994.

[5] C. R. Becker, A. Majeed, et al., “CT measurement of coronary calcium mass: impact on global cardiac risk assessment,” *European Radiology*, vol. 15, no. 1, pp. 96–101, 2005.

[6] J. J. Carr, J. C. Nelson, N. D. Wang, et al., “Calcified coronary artery plaque measurement with cardiac CT in population-based studies: standardized protocol of Multi-Ethnic Study of Atherosclerosis (MESA) and Coronary Artery Risk Development in Young Adults (CARDIA) study,” *Radiology*, vol. 234, no. 1, pp. 35–43, 2005.

[7] P. G. O’Malley, B. A. Greenberg, and A. J. Taylor, “Cost-effectiveness of using electron beam computed tomography to identify patients at risk for clinical coronary artery disease,” *American Heart Journal*, vol. 148, no. 1, pp. 106–113, 2004.

[8] K. Pohle, M. Otte, R. Mäffert, et al., “Association of cardiovascular risk factors to aortic valve calcification as quantified by electron beam computed tomography,” *Mayo Clinic Proceedings*, vol. 79, no. 10, pp. 1242–1246, 2004.

[9] A. Schmermund, S. Möhlenkamp, S. Berenbein, et al., “Population-based assessment of subclinical coronary atherosclerosis using electron-beam computed tomography,” *Atherosclerosis*, vol. 185, no. 1, pp. 177–182, 2006.

[10] S. Ulzheimer and W. A. Kalender, “Assessment of calcium scoring performance in cardiac computed tomography,” *European Radiology*, vol. 13, no. 3, pp. 484–497, 2003.

[11] A. T. Jones, D. M. Hansell, and T. W. Evans, “Quantifying pulmonary perfusion in primary pulmonary hypertension using electron-beam computed tomography,” *European Respiratory Journal*, vol. 23, no. 2, pp. 202–207, 2004.

[12] C. C. Morehouse, W. R. Brody, D. F. Guthaner, et al., "Gated cardiac computed tomography with a motion phantom," *Radiology*, vol. 134, no. 1, pp. 213–217, 1980.

[13] S. C. Moore, P. F. Judy, J. D. Garnic, et al., "Prospectively gated cardiac computed tomography," *Medical Physics*, vol. 10, no. 6, pp. 846–855, 1983.

[14] J. Horiguchi, T. Nakanishi, K. Ito, et al., "Initial experience of cardiac multidetector-row CT using a multisector reconstruction algorithm: studies of normal volunteers," *Nippon Igaku Hoshasen Gakkai Zasshi*, vol. 60, no. 12, pp. 705–706, 2000.

[15] W. R. Janowitz, "Current status of mechanical computed tomography in cardiac imaging," *American Journal of Cardiology*, vol. 88, no. 2A, pp. 35E–38E, 2001.

[16] A. F. Kopp, B. Ohnesorge, T. Flohr, et al., "Cardiac multidetector-row CT: first clinical results of retrospectively ECG-gated spiral with optimized temporal and spatial resolution," *RoFo Fortschritte auf dem Gebiet der Rontgenstrahlen und der Bildgebenden Verfahren*, vol. 172, no. 5, pp. 429–435, 2000.

[17] M. Kachelriess, S. Ulzheimer, and W. A. Kalender, "ECG-correlated imaging of the heart with subsecond multislice spiral CT," *IEEE Transactions on Medical Imaging*, vol. 19, no. 9, pp. 888–901, 2000.

[18] M. Kachelriess, D. A. Sennst, W. Maxlmoser, et al., "Kymogram detection and kymogram-correlated image reconstruction from subsecond spiral computed tomography scans of the heart," *Medical Physics*, vol. 29, no. 7, pp. 1489–1503, 2002.

[19] B. Ohnesorge, T. Flohr, C. Becker, et al., "Cardiac imaging by means of electrocardio-graphically gated multisection spiral CT: initial experience," *Radiology*, vol. 217, no. 2, pp. 564–571, 2000.

[20] K. Taguchi and H. Anno, "High temporal resolution for multislice helical computed tomography," *Medical Physics*, vol. 27, no. 5, pp. 861–872, 2000.

[21] G. Wang, S. Zhao, and D. Heuscher, "A knowledge-based cone-beam x-ray CT algorithm for dynamic volumetric cardiac imaging," *Medical Physics*, vol. 29, no. 8, pp. 1807–1822, 2002.

[22] K. Marten, M. Funke, M. Rummeny, et al., "Electrocardiographic assistance in multidetector CT of thoracic disorders," *Clinical Radiology*, vol. 60, no. 1, pp. 8–21, 2005.

[23] C. R. Crawford, K. F. King, C. J. Ritchie, and J. D. Godwin, "Respiratory compensation in projection imaging using a magnification and displacement model," *IEEE Transactions on Medical Imaging*, vol. 15, no. 3, pp. 327–332, 1996.

[24] N. Aggarwal, S. Bandyopadhyay, and Y. Bresler, "Spatio-temporal modeling and adaptive acquisition for cardiac MRI," in *Proccedings of IEEE International Symposium on Biomedical Imaging: Macro to Nano (ISBI '04)*, vol. 1, pp. 628–631, Arlington, Va, USA, April 2004.

[25] C. Ritchie, C. Crawford, J. Godwin, K. King, and Y. Kim, "Correction of computed tomography motion artifacts using pixel-specific back projection," *IEEE Transaction on Medical Imaging*, vol. 15, no. 3, pp. 333–342, 1996.

[26] M. W. Vannier and G. Wang, "Spiral CT refines imaging of temporal bone disorders," *Diagnostic imaging*, vol. 15, no. 11, pp. 116–121, 1993.

[27] G. Wang, Y. Liu, Y. Ye, et al., "Top-level design and preliminary physical analysis for the first electron-beam micro-CT scanner," *Journal of X-Ray Science and Technology*, vol. 12, no. 4, pp. 251–260, 2004.

[28] G. Wang and M. W. Vannier, "Micro-CT scanners for biomedical applications: an overview," *Advanced Imaging*, vol. 16, no. 7, pp. 18–27, 2001.

[29] C. Badea, L. W. Hedlund, and G. A. Johnson, "Micro-CT with respiratory and cardiac gating," *Medical Physics*, vol. 31, no. 12, pp. 3324–3329, 2004.

[30] A. Katsevich, "Theoretically exact filtered backprojection-type inversion algorithm for spiral CT," *SIAM Journal on Applied Mathematics*, vol. 62, no. 6, pp. 2012–2026, 2002.

[31] A. Katsevich, "An improved exact filtered backprojection algorithm for spiral computed tomography," *Advances in Applied Mathematics*, vol. 32, no. 4, pp. 681–697, 2004.

[32] X. Tang and J. Hsieh, "A filtered backprojection algorithm for cone beam reconstruction using rotational filtering under helical source trajectory," *Medical Physics*, vol. 31, no. 11, pp. 2949–2960, 2004.

[33] X. Tang, J. Hsieh, A. Hagiwara, et al., "A three-dimensional weighted cone beam filtered backprojection (CB-FBP) algorithm for image reconstruction in volumetric CT under a circular source trajectory," *Physics in Medicine and Biology*, vol. 50, no. 16, pp. 3889–3905, 2005.

[34] G. Wang, T. H. Lin, P.-C. Cheng, et al., "General cone-beam reconstruction algorithm," *IEEE Transactions on Medical Imaging*, vol. 12, no. 3, pp. 486–496, 1993.

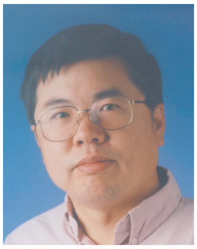

**Erwei Bai** was educated in Fudan University, and Shanghai Jiaotong University, both in Shanghai, China, and the University of California at Berkeley. He is a Professor of electrical and computer engineering at the University of Iowa where he teaches and conducts research in identification, control, and their applications in medicine and communication. He is an IEEE Fellow.

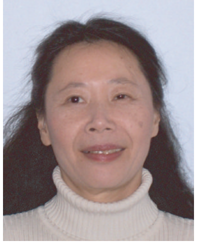

**Chenglin Wang** was educated in Shanghai Jiaotong University, Shanghai, China, and the University of Pittsburgh, Pittsburgh, Pennsylvania, both in electrical engineering. She have been working as an engineer for various small companies, specialized in design and testing. Her current interests lie in medical imaging reconstruction and analysis. She has been with Controlled Cardiac CT since 2005.

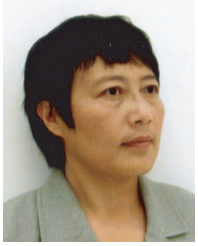

**Ying Liu** received the B.E. degree in telecommunications from Xidian University (Xian, China) in 1982, the M.S. degree and the Ph.D. degree in electrical and computer engineering from the State University of New York (Buffalo, New York) in 1990 and 1995, respectively. She worked as a system Engineer with Voice Technologies Group (Buffalo, New York) from 1990 to 1991, a system Engineer with Electronic Data Systems (St. Louis, Missouri) from 1994 to 1996, a Programmer Analyst with Impact Technologies (St. Louis, Missouri) from 1996 to 1997, a Consultant with Rockwell Collins (Cedar Rapids, Iowa) from 1997 to 1998, a Software Developer with National Computer Systems (Iowa City, Iowa) since 1998, and also a part-time Senior Software Engineer with Controlled Cardiac CT (Iowa City, Iowa) since 2005. She has been working on digital imaging, image reconstruction, analysis and visualization, software development, and project management. She published 7 scientific papers.

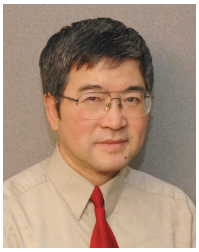

**Ge Wang** received the B.E. degree in electrical engineering from Xidian University, Xian, China, in 1982, the M.S. degree in remote sensing from Graduate School of Academia Sinica, Beijing, China, in 1985, and the M.S. degree and the Ph.D. degree in electrical and computer engineering from the State University of New York, Buffalo, in 1991 and 1992. He was an Instructor and Assistant Professor with the Department of Electrical Engineering, Graduate School of Academia Sinica, in 1984–1988, an Instructor and Assistant Professor with Mallinckrodt Institute of Radiology, Washington University, St. Louis, Mo, in 1992–1996. He was an Associate Professor with University of Iowa from 1997 to 2002. Currently, he is a Professor with the Departments of Radiology, Biomedical Engineering, Civil Engineering, and Mathematics, and the Director of the Center for X-ray and Optical Tomography, University of Iowa. His research interests include computed tomography, bioluminescence tomography, and systems biomedicine. He has authored or coauthored over 300 journal articles and conference papers, including the first paper on spiral/helical cone-beam CT and the first paper on bioluminescence tomography. He is the Editor-in-Chief for the International Journal of Biomedical Imaging, and an Associate Editor for the IEEE Transactions on Medical Imaging and Medical Physics. He is an IEEE Fellow and an AIMBE Fellow. He is also recognized by a number of awards for academic achievements.

